# Optimization-Driven Engineering of Electrodeposited Nanographenide–Conductive Polymer/Prussian Blue Nanoarchitectures for Robust Electrochemical Sensing

**DOI:** 10.3390/s26082427

**Published:** 2026-04-15

**Authors:** Seung Joo Jang, Hong Chul Lim, Tae Hyun Kim

**Affiliations:** 1 Department of Chemistry, Soonchunhyang University, Asan 31538, Republic of Korea; seen813@sch.ac.kr; 2 Department of Pharmaceutics and Biopharmacy, Sangji University, Wonju 26339, Republic of Korea

**Keywords:** nanographenide, nanoarchitecture, electrode active material, electrodeposition, electrochemical sensors

## Abstract

The development of high-performance electrochemical sensors requires precise integration of electrode active materials that provide both superior electrocatalytic activity and long-term structural stability. Herein, we report a systematically optimized, one-pot electrochemical deposition approach for the fabrication of nanographenide-based nanoarchitectures, incorporating either a conducting polymer (PEDOT-NG) or Prussian blue (PB-NG). Derived from optimization-driven structural refinement—including applied potential, electrodeposition time, and precursor concentration—the robust nanoarchitecture exhibits a hierarchical morphology that provides an expanded electroactive surface area, accelerating charge transfer and enhancing electrochemical catalytic activity. The optimized PEDOT-NG exhibits exceptional sensitivity for the simultaneous determination of ascorbic acid (AA), dopamine (DA), and uric acid (UA), achieving wide linear ranges with low detection limits of 4.1, 0.12, and 0.18 μM, respectively. The PB-NG achieves a limit of detection of 4.39 μM, driven by highly reversible and stable redox kinetics. This performance is underpinned by narrowed peak-to-peak separations (ΔE) and reduced redox potentials. These results underscore the pivotal role of precise parametric control in developing high-performance electrochemical sensors. Furthermore, this work establishes a comprehensive strategy for designing resilient electrode active materials, thereby paving the way for next-generation electrochemical platforms tailored for diverse and robust sensing environments.

## 1. Introduction

The demand for high-performance electrochemical sensors in clinical diagnostics and environmental monitoring has intensified the search for electrode active materials with superior sensitivity, selectivity, and long-term stability [1,2,3]. Electrode active materials are critical components of these sensors, serving a dual role as both the recognition element for molecular detection and the transducer for signal transmission [1,4]. Nanostructured hybrid materials have emerged as promising candidates due to their ability to yield synergistic electrochemical catalytic effects unattainable by individual components [5,6,7]. Through the rational design of nanoarchitectures, the intrinsic strengths of each constituent can be strategically exploited to lower detection limits (LOD) and enhance the sensitivity of electrochemical sensors [8,9,10]. In this regard, significant attention has been directed toward nanoarchitectures that integrate noble metal nanoparticles (NPs) with electroactive species such as Prussian blue (PB) or poly(3,4-ethylenedioxythiophene) (PEDOT). The PB is widely recognized as an artificial peroxidase that serves as a highly efficient electrocatalyst for H_2_O_2_ reduction; its practical application is often hampered by several inherent limitations. Firstly, the intercalation of counter-ions during the redox reactions of PB induces significant volume changes and mechanical stress, leading to structural instability [11]. Secondly, Inhomogeneous electrical conductivity across the PB lattice often induces parasitic side reactions, which detrimentally affect the material’s electrochemical stability and performance [12]. Furthermore, PB is prone to decomposition under neutral or alkaline conditions (pH > 7), which limits its utility in physiological environments [13]. The intrinsically low electrical conductivity of PEDOT and its volume fluctuations—caused by swelling and shrinking during redox processes—often lead to sluggish electron transfer and signal drift [14]. These limitations hinder the realization of high sensitivity and long-term operational stability [15]. Although various fabrication methods for nanoarchitectures have been explored, achieving stable, uniform integration of NPs with electrode active materials such as PB and PEDOT remains a persistent challenge. Most conventional techniques rely on multi-step processes that are not only time-consuming but also poorly reproducible, thereby driving a growing demand for more cost-effective and structurally robust alternatives [16,17,18]. Furthermore, the practical realization of conventional nanoarchitectures is frequently hindered by the inherent trade-offs between electrochemical performance and fabrication feasibility [19]. Addressing these constraints requires an integrated approach that simultaneously optimizes electron- and ion-transport morphologies within the nanoarchitecture, while ensuring the simplicity and scalability of the fabrication process. Our previous studies have demonstrated the feasibility of NG-based nanoarchitectures, such as PB–NG and PEDOT–NG, for electrochemical sensing applications [4,20]. However, those studies primarily focused on demonstrating sensing capability using individual nanoarchitectures. A systematic investigation of the fabrication parameters governing the structural formation and electrocatalytic performance of NG-based hybrid electrodes has not yet been reported.

In this work, we focus on optimization-driven engineering of electrodeposited NG-based nanoarchitectures by systematically investigating key electrodeposition parameters, including potential window, deposition cycles, and solution pH. This approach enables us to establish clear process–structure–performance relationships for robust electrochemical sensing platforms. The resulting robust NG-based nanoarchitecture exhibits a hierarchical morphology that significantly expands the electroactive surface area, thereby accelerating heterogeneous electron transfer and enhancing electrochemical catalytic activity. This performance is underpinned by narrowed peak-to-peak separations (ΔE) and reduced redox potentials, driven by highly reversible and stable kinetics. Specifically, the optimized PEDOT-NG platform demonstrates exceptional sensitivity for the simultaneous determination of ascorbic acid (AA), dopamine (DA), and uric acid (UA), achieving low detection limits of 4.1, 0.12, and 0.18 μM, respectively. Furthermore, the PB-NG architecture achieves a detection limit of 4.39 μM, highlighting the efficacy of our precise parametric control. These results underscore a comprehensive strategy for designing resilient electrode active materials, paving the way for next-generation electrochemical sensing tailored to diverse and robust sensing environments.

## 2. Materials and Methods

### 2.1. Chemicals and Reagents

All the chemical reagents used in this study were commercially available and used without further purification. Carbon nanofiber, sulfuric acid (H_2_SO_4_), potassium carbonate (K_2_CO_3_), ascorbic acid (AA), dopamine hydrochloride (DA), uric acid (UA), glycine, D-(+)-glucose, L-cysteine, and phosphate-buffered saline (PBS, pH 7.4, 0.1 M) were purchased from Sigma-Aldrich (St. Louis, MO, USA). hydrogen peroxide (H_2_O_2_), iron(III) chloride hexahydrate (FeCl_3_⋅6H_2_O), potassium ferrocyanide (K_4_[Fe(CN)_6_]), and potassium ferricyanide (K_3_[Fe(CN)_6_]) were purchased from ThermoFisher (Waltham, MA, USA). Nitric acid (HNO_3_), ethanol, acetonitrile (CH_3_CN), and potassium chloride (KCl) were purchased from Daejung Chemical & Metals Co., Ltd. (Siheung, Republic of Korea). Screen-printed electrodes (SPEs) (working electrode: 4 mm diameter Pt, counter electrode: Pt, and reference electrode: Ag) were purchased from Metrohm DropSens (Metrohm AG, Herisau, Switzerland).

### 2.2. Preparation of KNG

The KNG was prepared by adding 1.5 g of carbon nanofibers to a 3:1 (*v*/*v*) mixture of concentrated H_2_SO_4_ (30 mL) and HNO_3_ (10 mL). The mixture was ultrasonicated for 1 h and subsequently stirred at 110 °C for 24 h. Upon cooling, the solution was treated with saturated K_2_CO_3_ to yield the KNG product. For purification and size fractionation, the crude mixture was centrifuged at 10,000 rpm for 30 min. Size-selective precipitation was achieved by incrementally adding ethanol to the supernatant at a 1:1 (*v*/*v*) ratio, followed by repeated centrifugation steps. This procedure was performed iteratively to isolate uniform KNG particles and eliminate undesired by-products [13].

### 2.3. One-Pot Electrodeposition Process of PB-NG and PEDOT-NG Nanoarchitecture

The PB-NG nanoarchitecture is prepared using chronoamperometry. This electrodeposition process took place either on screen-printed electrodes (SPEs) or within a three-electrode system comprising a reference electrode (Ag/AgCl), a counter electrode (Pt wire), and a working electrode (ITO) in an aqueous solution containing 0.05 M K_3_[Fe(CN)_6_], 0.05 M FeCl_3_⋅6H_2_O, and KNG. The PEDOT-NG nanoarchitecture is fabricated on SPEs via repetitive cyclic voltammetry (CV). The electrochemical cells contain EDOT as a monomer and KNG as a supporting electrolyte and anion dopant in H_2_O:CH_3_CN (1:9 *v*/*v*) mixture system. After the electrodeposition process, all samples were rinsed with DI water or CH_3_CN to remove residual precursors and impurities and dried in an oven at 100 °C for 10 min.

### 2.4. Electrochemical Characterization of Electrodeposited PB-NG and PEDOT-NG Nanoarchitecture Thin Films

The electrochemical behaviors of PB-NG and PEDOT-NG nanoarchitecture were evaluated in 0.01 M PBS buffer solution under a three-electrode system (a reference electrode: Ag/AgCl, a counter electrode: Pt wire, and a working electrode: PB-NG or PEDOT-NG nanoarchitecture). The electrochemical catalytic activity of PB-GQD nanoarchitecture toward H_2_O_2_ was evaluated using chronoamperometry in a 0.01 M PBS (pH 4.0) buffer solution containing 0.1 M KCl. The measurements were conducted at a constant applied potential of 0.00 V (vs. Ag/AgCl). The electrochemical sensing of the PEDOT-NG nanoarchitectures was performed using differential pulse voltammetry (DPV) in a 0.1 M PBS (pH 7.4) solution containing AA, DA, and UA. The DPV measurements were conducted by scanning the potential in the positive direction from −0.2 V to 0.6 V, with an increment E of 0.006 V and an amplitude of 0.04 V. All electrochemical experiments were carried out at room temperature without removing dissolved oxygen from the solution.

### 2.5. Instruments

The electrochemical experiments were performed with a CHI instrument (electrochemical analyzer, CHI 660D (CHI Instrument, Inc., Austin, TX, USA). Electrochemical impedance spectroscopy (EIS) was performed in 10 mM K_4_Fe(CN)_6_ with 1.0 M KCl at 0 V, from 100 kHz to 0.01 Hz with an AC amplitude of 10 mV. UV-Vis-NIR absorption spectra (JASCO, V-670, Hachioji, Tokyo, Japan) were obtained to confirm the optical properties of the as-prepared thin films. The surface morphologies of thin films were obtained by a field-emission scanning electron microscope (FE-SEM) (Hitachi, S-4300, Chiyoda, Japan). The chemical composition of PB-NG nanoarchitecture was analyzed by X-ray photoelectron spectroscopy (XPS) (Kratos, AXIS Supra, Shimadzu, Kyoto, Japan) using a monochromatic Alkα (15 kV, 375 W). The thickness of electrodeposited thin films was measured using a Dektak XT-A stylus profilometer (Bruker, Germany). Raman spectroscopy (EnSpectr R532, Enhanced Spectrometry, Inc., San Jose, CA, USA) was conducted with a 633 nm Ar-ion laser to check vibrational modes of the PEDOT structure in as-prepared thin films.

## 3. Results and Discussion

### 3.1. One-Pot Electrodeposition Mechanism and Optimization of Fabrication Parameters for PB-NG Nanoarchitecture Thin Film

**Scheme 1:**
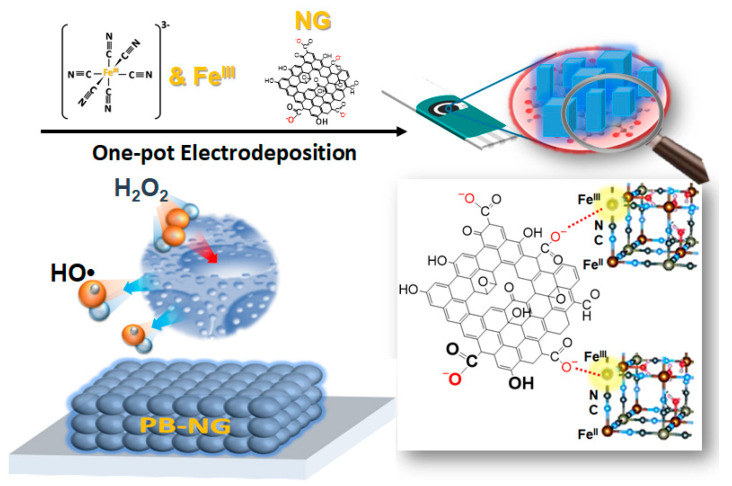
Schematic illustration of PB-NG nanoarchitecture thin film through one-pot electrochemical deposition process.

The plausible electrodeposition mechanism for the PB-NG nanoarchitecture on electrode substrates (e.g., ITO and SPE) is illustrated in Scheme 1. When a positive potential is applied to the working electrode in the initial stage, Fe(CN)_6^3−^_ ions migrate to the electrode surface and undergo electrostatic attraction or coordination bonding with NG^n−^ as nucleation site. Subsequently, Fe(CN)_6^3−^_ ions undergo reduction via heterogeneous electron transfer mediated by the NG on the electrode surface at an applied potential. In the following stage, robust ionic and covalent bonds form between Fe^3+^ ions dissociated from FeCl_3_ and the newly reduced Fe(CN)_6^4−^_ species, a process driven by the principles of the Hard and Soft Acids and Bases theory [21]. This interaction triggers the formation of coordinated Fe(III)-N-C-Fe(II) complexes on both the basal planes and edges of the NG^n−^, thereby establishing a stable, integrated PB-NG nanoarchitecture. Based on the proposed reaction mechanism, the performance of the PB-NG nanoarchitecture thin films was systematically evaluated by modulating key fabrication parameters, including the applied potential, precursor concentration, and electrodeposition time.

**Figure 1:**
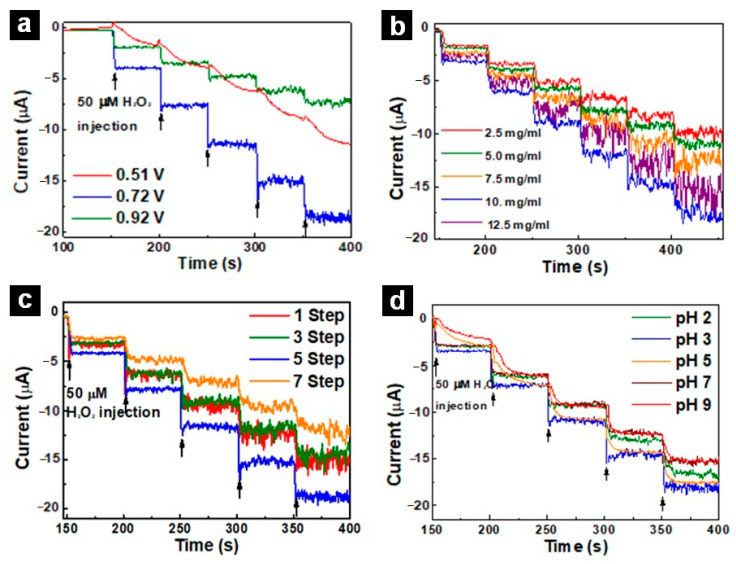
Amperometric responses of PB-NG nanoarchitecture thin films upon the successive addition of 50 μM H_2_O_2_ in 0.01 M PBS (pH 4) buffer solution containing 0.1 M KCl at an applied potential of 0.00 V (vs. Ag/AgCl). Comparison of the response currents as a function of (**a**) electrodeposition potential, (**b**) KNG concentration, (**c**) electrodeposition duration, and (**d**) pH condition during the electrochemical deposition process.

The effect of the potential applied to the working electrode, ranging from 0.51 V to 0.92 V (vs. Ag/AgCl), was investigated to determine its influence on the PB-NG nanoarchitecture. The applied potential during electrodeposition is a critical factor governing the nucleation density and growth rate of the nanoarchitecture, which directly dictates its resulting electrochemical performance [4,22]. Figure 1a shows the amperometric responses of the PB-NG nanoarchitecture thin films, fabricated at various electrodeposition potentials. The electrochemical catalytic activity of PB-NG nanoarchitecture thin films toward H_2_O_2_ reduction was evaluated by monitoring current response at an applied potential of 0.00 V (vs. Ag/AgCl) in a 0.01 M PBS (pH 4.0) buffer with successive additions of 50 μM H_2_O_2_. Because the reduction of PB NPs involving high-spin iron signals yields a superior amperometric response, whereas the oxidation process associated with low-spin iron signals exhibits sluggish kinetics and poor electrochemical stability [20,23]. The electrode prepared at 0.72 V exhibited the most pronounced and well-defined current steps with the highest magnitude, indicating superior electrocatalytic activity and sensitivity. In contrast, the sample fabricated at 0.51 V exhibited an unstable, sluggish response with significant baseline drift, likely due to insufficient and non-uniform formation of the PB-NG active layer. Furthermore, the 0.92 V sample displayed a noticeably reduced current response compared to the 0.72 V counterpart. This performance attenuation at higher potentials can be attributed to uncontrolled aggregation of PB NPs, which diminishes the effective electroactive surface area [14,24]. Consequently, 0.72 V was identified as the optimal electrodeposition potential for achieving the most robust and sensitive sensing interface.

The influence of KNG concentration on the electrochemical catalytic performance of the PB-NG nanoarchitecture was evaluated toward the determination of H_2_O_2_. As shown in Figure 1b, the amperometric *i-t* curves response increased with KNG concentration up to 10 mg mL^−1^, reaching a maximum sensitivity. This enhancement is attributed to the increased surface area of the NG scaffold, which facilitates the dense, stable integration of PB active sites. However, at a higher concentration of 12.5 mg mL^−1^, a noticeable decrease in current magnitude and increased background noise were observed, likely due to the aggregation of PB nanostructure and hindered mass transport at the electrode interface. Consequently, 10 mg mL^−1^ was determined as the optimal KNG concentration for robust H_2_O_2_ determination.

The PB-NG nanoarchitecture was fabricated by varying the electrodeposition duration at a constant KNG concentration of 10 mg mL^−1^ with an applied potential of 0.72 V (vs. Ag/AgCl). Following the repetitive potential step profile shown in Figure S1, each cycle involved a 50 s pulse within the potential range of 0.72–0.77 V (vs. Ag/AgCl). The amperometric *i-t* curves response toward 50 μM H_2_O_2_ significantly improved as the number of steps increased from 1 to 5, as shown in Figure 1c. The PB-NG nanoarchitecture thin films with 5 cycles exhibited the highest electrochemical catalytic current and the most stable baseline, indicating the formation of an optimal hierarchical nanoarchitecture with maximized active sites, as depicted in Figure S2. However, further increasing the deposition to 7 cycles resulted in a marked decline in sensitivity. The sensing performance of the electrodeposited nanoarchitectures is strongly influenced by the structural characteristics formed during the electrodeposition process. In particular, the deposition cycle number plays a critical role in determining the morphology and catalytic site density of the NG-based nanoarchitectures. At low deposition cycles, only a limited number of catalytic nanoparticles are nucleated on the NG scaffold, resulting in a relatively small electroactive surface area and weak electrochemical responses. As the deposition cycles increase, catalytic clusters become more densely distributed along the conductive NG network, which enhances the electrochemically active surface area and facilitates efficient electron transfer. However, excessive deposition cycles may lead to the formation of thicker or aggregated structures that partially block active sites and hinder mass transport of analyte molecules [22,25]. Therefore, an optimal deposition condition is required to achieve a balanced nanoarchitecture with abundant catalytic sites and efficient electron transport pathways, ultimately leading to improved sensing performance. Consequently, a total of 5 cycles of electrodeposition was selected as the optimal deposition time for the fabrication of the PB-NG sensor.

The pH of the electrodeposition solution plays a critical role in the formation and structural integrity of PB-NG nanoarchitectures. The electrodeposition process was systematically conducted across a range of pH conditions, while maintaining a constant KNG concentration of 10 mg mL^−1^ and an applied potential of 0.72 V (vs. Ag/AgCl) for 5 cycles. Figure S3 presents the FE-SEM images of the PB-NG nanoarchitecture thin films electrodeposited at various pH conditions. The morphology of the PB-NG nanoarchitecture thin films was remarkably dependent on the pH of the precursor solution. At the optimal pH of 3.0, the PB-NG exhibited a highly dense, smooth, and uniform surface without any visible defects. In contrast, at pH 2.0, a slightly less packed structure was observed, and as the pH increased further toward neutrality (pH 7.0) and alkalinity (pH 9.0), the morphology was observed as significantly cracked and rough surfaces with irregularly sized clusters. The observed morphological change is likely attributed to the structural degradation of the Fe(III)-C-N-Fe(II) backbone, which occurs via the nucleophilic attack of OH^−^ ions, which triggers the formation of iron hydroxide (Fe(OH)_3_) at higher pH levels. This chemical instability leads to the fragmented and cracked morphology observed in the PB-NG nanoarchitecture [13,20]. As shown in Figure 1d, the amperometric response to successive 50 μM H_2_O_2_ injections was evaluated at 0.01 M PBS (pH 4) buffer solution. Among the various pH conditions evaluated, the precursor solution at pH 3.0 proved to be the most effective, yielding a PB-NG nanoarchitecture with a superior amperometric response. This optimized film was characterized by well-defined, discrete steps and a rapid steady-state response upon each addition of H_2_O_2_. Conversely, as the pH increased precursor solution toward neutral (pH 7.0) and alkaline (pH 9.0) conditions, the current response significantly decreased, and the neutrality (pH 7.0) and alkalinity (pH 9.0), the current response decreased significantly, and baseline stability deteriorated. The pH-dependent growth behavior of PB-NG nanoarchitecture suggests that the acidity of the precursor solution modulates the nucleation rate and the subsequent assembly of PB onto the NG template. This systematic approach allowed for the controlled assembly of the nanoarchitecture by regulating nucleation and growth kinetics, which proved essential for achieving robust H_2_O_2_ sensing performance.

### 3.2. Characterization of PB-NG Nanoarchitecture Thin Film

**Figure 2:**
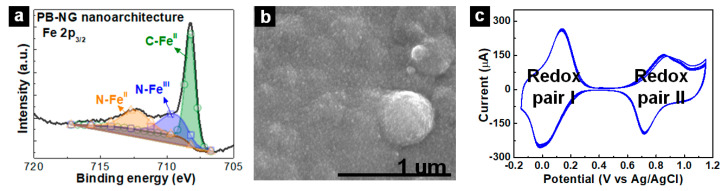
Characterization of optimized PB-NG nanoarchitecture. (**a**) XPS narrow spectrum of Fe 2p. (**b**) SEM image. (**c**) Cyclic voltammogram in 0.01 M PBS (pH 4) solution with 0.1 M KCl at 50 mV s^−1^.

To further elucidate the chemical composition and oxidation states of iron within the PB-NG nanoarchitecture, XPS analysis was performed. Regarding the Fe 2p_3/2_ core-level spectrum, the signal was successfully deconvoluted into three distinct peaks as shown in Figure 2a. The most prominent peak at 708.3 eV is attributed to the low-spin Fe^2+^ species coordinated to carbon atoms C-Fe(II) within the [Fe(CN)6]^4−^ units. The peaks observed at 709.6 eV and 712.5 eV correspond to the high-spin Fe^3+^ (Fe(III)) and low-spin Fe^2+^ (Fe(II)) species coordinated to nitrogen atoms, respectively [20,26]. These binding energy values are consistent with the characteristic coordination environment of PB, confirming that the PB NPs are robustly integrated onto the NG surface while maintaining a unique mixed-valence electronic structure. The UV-Vis spectrum of the PB-NG nanoarchitecture on ITO/glass electrode substrate exhibits a broad absorption band centered at approximately 700 nm, as shown in Figure S4, which is attributed to the metal-to-metal charge transfer between Fe^2+^ and Fe^3+^ through the cyano-bridges in the face-centered cubic structure of PB [27]. The surface morphology of the optimized PB-NG nanoarchitecture was characterized using SEM, as displayed in Figure 2b. The PB-NG nanoarchitecture exhibits a hierarchical granular structure, with small PB NPs forming across the NG surface. Some larger spherical clusters with several hundred nanometers in size are also observed. The electrochemical behavior of the PB-NG nanoarchitecture thin films, prepared under the optimized fabrication conditions, was evaluated on the ITO/glass electrode substrate using CV in a 0.01 M PBS (pH 4) solution containing 0.1 M KCl at a scan rate of 50 mV s^−1^. The resulting CV curve exhibits two well-defined redox couples, with half-wave potentials (E_1/2_ = (E_pa_ + E_pc_)/2) of 0.073 V and 0.793 V (vs. Ag/AgCl) for redox pairs I and II, respectively, as shown in Figure 2c. Redox pair I, spanning from 0.3 V to −0.15 V, is attributed to the Fe^3+^/^2+^ species coordinated to nitrogen atoms [20]. This transition represents the conversion between Prussian blue (PB) and Prussian white (PW), which can be described by Equation (1) [20,28]:

(1) $$\left(\mathrm{PB})\;{Fe}_{4}^{III}{\left[{Fe}^{II}{\left(CN\right)}_{6}\right]}_{3}+4K^{+}+4e^{-}⇄K_{4}{Fe}_{4}^{II}{\left[{Fe}^{II}{\left(CN\right)}_{6}\right]}_{3}\;(PW\right)$$

Redox pair II, observed between 0.7 and 1.1 V, is associated with the low-spin state of Fe^2+^/^3+^ species coordinated to carbon atoms. This electrochemical process represents the reversible transition between Prussian blue (PB) and Berlin green (BG), which involves the extraction/insertion of anions to maintain charge neutrality. This transition can be expressed by Equation (2):

(2) $$\left(\mathrm{PB})\;{Fe}_{4}^{III}{\left[{Fe}^{II}{\left(CN\right)}_{6}\right]}_{3}+4{Cl}^{-}⇄K_{4}{Fe}_{4}^{III}{\left[{Fe}^{III}{\left(CN\right)}_{6}Cl\right]}_{3}+3e^{-}\;(BG\right)$$

The relatively small peak-to-peak separation (ΔE_p_) and a peak current ratio (I_pa_/I_pc_) close to unity indicate facilitated heterogeneous electron transfer kinetics and highly reversible counter-cation (e.g., K^+^) intercalation/deintercalation within the PB-NG interfacial nanoarchitecture. The PB-NG nanoarchitecture thin films are proportional to the square root of the scan rate (*v*), as shown in Figure S5. Therefore, redox reactions of the thin film depend on the insertion and desertion of counter ions (K^+^ ions) into the inside of the thin film to maintain the charge balance of the PB, which is controlled by the diffusion of counter ions. NG plays a pivotal role not only in promoting the precise and uniform growth of PB NPs but also in serving as a template that guides their formation through coordination interactions and electrostatic attraction with iron cations—the essential precursors of PB. Consequently, the PB-NG nanoarchitecture provides abundant electrochemically active sites and shortened mass transfer distances, effectively alleviating structural strain caused by volume expansion. The electrocatalytic response of the PB-NG nanoarchitecture thin films toward H_2_O_2_ detection exhibited high reproducibility across all five tested electrodes, as illustrated in Figure S6. In contrast to conventional studies focused on PB-based composites, the proposed PB-NG nanoarchitecture specifically targets PB’s unique electrocatalytic properties for the determination of H_2_O_2_. This design strategy facilitates superior sensing capabilities, including a broad linear range and a low LOD, as detailed in the comparative summary in Table S1.

### 3.3. One-Pot Electrodeposition Mechanism and Optimization of Fabrication Parameters for PEDOT-NG Nanoarchitecture Thin Film

**Scheme 2:**
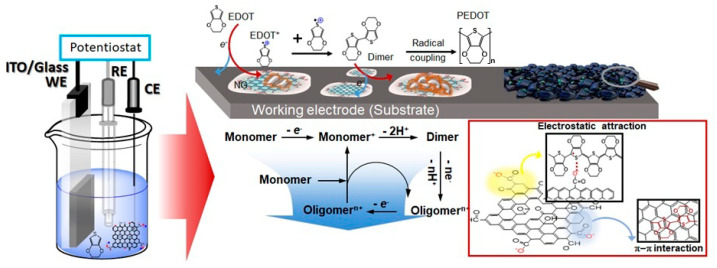
Schematic illustration of PEDOT-NG nanoarchitecture thin film through one-pot electrochemical deposition process.

Scheme 2 illustrates the electrodeposition process of PEDOT-NG nanoarchitecture thin films onto various electrode substrates, such as ITO and SPE. Notably, KNG plays a multifunctional role, acting as both a supporting electrolyte to facilitate ionic conductivity and an anion dopant to induce conformational changes in the PEDOT structure [4,14]. Raman spectroscopy provides critical insights into the polymer’s chain conformation and doping interactions. Compared to conventional PEDOT thin films, the PEDOT-NG nanoarchitecture exhibits a red shift of 17 cm^−1^ in the C_α_ = C_β_ symmetric stretching mode, as depicted in Figure S7. This red shift is indicative of the successful integration of NG^n−^ and suggests a structural transition toward a quinoid state, which is likely associated with enhanced π-electron delocalization and improved electrical conductivity [4]. During the process, the nano-sized NG is incorporated into the oxidized PEDOT backbone via electrostatic attraction and π-π interactions. The NG serves as a functional template that regulates the nucleation and growth of PEDOT, effectively suppressing excessive aggregation. This results in a conformational change in the PEDOT backbone, creating an expanded-coil quinoid structure and an interconnected three-dimensional (3D) network with a large effective surface area, which facilitates efficient charge transfer across the electrode interface. The electrochemical sensing performance of the PEDOT-NG nanoarchitecture for the simultaneous determination of biomolecules, including AA, DA, and UA, was systematically evaluated as a function of electrodeposition conditions.

**Figure 3:**
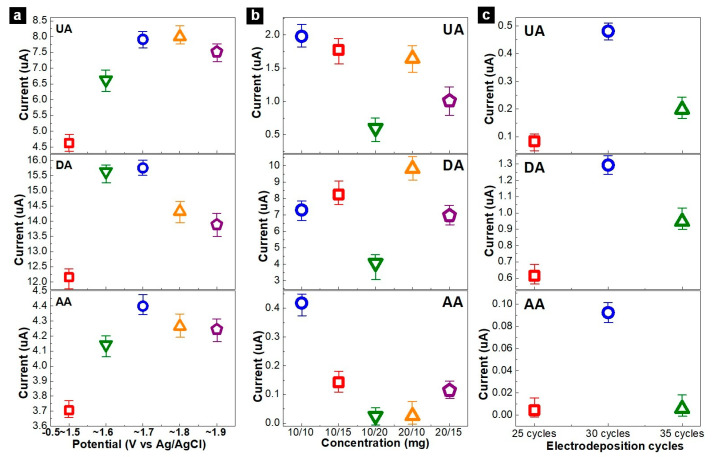
DPV cures of PEDOT-NG nanoarchitecture thin films upon 50 μM AA, 10 μM DA, and 10 μM UA with different electrodeposition conditions in 0.1 M PBS (pH 7.4) buffer solution. Comparison of the response currents as a function of (**a**) electrodeposition potential, (**b**) concentration of KNG and EDOT, and (**c**) electrodeposition cycles.

For the simultaneous determination of AA, DA, and UA, the electrodeposition potential was optimized, and the obtained DPV response curves are presented in Figure 3a. The oxidation current densities for all three biomolecules significantly increased as the electrodeposition potential was scanned from 1.5 V to 1.9 V (vs. Ag/AgCl). Specifically, the PEDOT-NG nanoarchitecture fabricated at 1.7 V exhibited the highest current responses for AA (4.4 μA), DA (15.8 μA), and UA (7.9 μA), indicating superior electrochemical catalytic activity. However, a further increase in the electrodeposition potential to 1.8 V and 1.9 V resulted in a gradual decrease in the current responses for AA and DA, while the response for UA remained relatively saturated. This performance decline at higher potentials is likely attributed to the overoxidation of the PEDOT backbone [4,29]. Applying an excessively high electrodeposition potential can trigger the irreversible overoxidation of the conjugated PEDOT backbone. This process degrades the conjugated system, significantly reducing the electronic conductivity of the polymer film and diminishing its electrochemical catalytic activity, as depicted in Figure S8. Based on these findings, 1.7 V was determined to be the optimal electrodeposition potential for fabricating the PEDOT-NG nanoarchitecture. The influence of precursor concentration ratios (EDOT/KNG) on the sensing performance toward AA, DA, and UA was systematically examined to identify the optimal composition for the PEDOT-NG nanoarchitecture thin films. As shown in Figure 3b, the amperometric responses varied considerably depending on the mass ratio of the precursors. The 10/10 mg ratio showed the highest and most balanced electrochemical catalytic activity, especially producing the maximum oxidation currents for AA (0.42 μA) and UA (1.98 μA). Although the 20/10 mg condition resulted in a slightly higher DA response (9.8 μA), the DPV curves exhibited a nearly negligible response to AA, which is problematic for multi-analyte sensing. This variation indicates that the stoichiometric balance between the monomer (EDOT) and the NG is pivotal in regulating the porosity of the interconnected 3D network and the density of electrochemically active sites. An excess or deficiency in either component can lead to uncontrolled aggregation or insufficient doping, thereby hindering effective charge transfer. Therefore, the 10/10 mg ratio was chosen as the optimal precursor concentration for further sensing experiments. The number of electrodeposition cycles was optimized to control the thickness and morphology of the PEDOT-NG thin films, while maintaining a constant KNG/EDOT concentration of 10/10 mg and an applied potential of 1.7 V (vs. Ag/Ag(NO)_3_). The response currents for AA, DA, and UA were highly dependent on the number of deposition cycles, with 30 cycles yielding the maximum sensing current for all three analytes, as shown in Figure 3c. The initial increase in current from 25 to 30 cycles is attributed to the formation of a higher density of electrochemically active sites in the PEDOT-NG nanoarchitecture thin film. However, beyond 30 cycles (35 cycles), a significant decrease in response current was observed. This performance degradation is likely due to excessive nanoarchitecture layer growth, which can clog internal pores and increase mass transfer resistance. Furthermore, overly thick films may suffer from reduced structural stability and poor adhesion to the electrode surface [30,31]. Consequently, 30 cycles were identified as the optimal electrodeposition duration for achieving the highest sensitivity in simultaneous detection of target molecular species. The average thickness of the PEDOT-NG nanostructured thin film obtained under optimal conditions was measured to be 1120 ± 20 nm.

**Figure 4:**
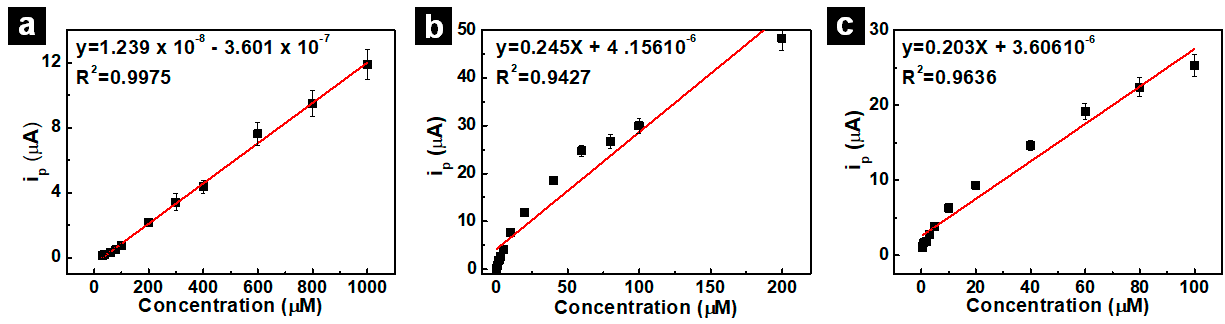
The current response for the simultaneous electrochemical detection of biomolecules using the optimized PEDOT-NG nanoarchitecture. The linear relationship between the peak oxidation current (*I*_pa_) and the concentration of (**a**) AA, (**b**) DA, and (**c**) UA. All measurements were performed using DPV in 0.1 M PBS (pH 7.4).

The quantitative sensing performance of the optimized PEDOT-NG nanoarchitecture was evaluated by monitoring the amperometric responses toward varying concentrations of AA, DA, and UA. As shown in Figure 4, the peak oxidation currents (I_pa_) exhibited a highly linear relationship with the analyte concentrations over wide ranges: 25–1000 μM for AA (R^2^ = 0.9975), 1–200 μM for DA (R^2^ = 0.9427), and 1–100 μM for UA (R^2^ = 0.9636). The calculated low detection limits, derived from the slopes of the calibration curves, were 4.1 μM for AA, 0.12 μM for DA, and 0.18 μM for UA. The LOD values were calculated using a signal-to-noise ratio of 3 to ensure their reliability.

LOD = (3 × σ)/S

where σ is the standard deviation of the response of the blank solution (background signal). *S* is the slope of the calibration curve in the dynamic range.

**Figure 5:**
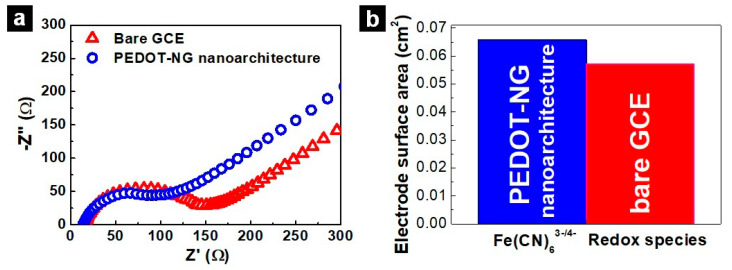
(**a**) Nyquist plots of 10 mM K_4_Fe(CN)_6_ in 1.0 M KCl aqueous solution and (**b**) calculated electrochemically active surface area.

To evaluate electron-transfer kinetics, the charge-transfer resistance (*R*_ct_) was determined. The obtained R_ct_ value for PEDOT-NG (99 Ω) was lower than that of the bare glassy carbon electrode (GCE) (130 Ω), indicating a more efficient electron-transfer process at the PEDOT-NG nanoarchitecture, as shown in Figure 5a. The electrochemically active surface area of the PEDOT-NG-modified electrode showed a significant 146% increase compared to that of the bare GCE, as depicted in Figure 5b, confirming that the hierarchical assembly of PEDOT and NG effectively expanded the number of available active sites for redox reactions. The remarkable electrochemical performance can be attributed to the hierarchical interconnected 3D network of the PEDOT-NG nanoarchitecture, which provides a high density of electroactive sites and facilitates rapid electron transfer at the electrode-electrolyte interface. The selectivity of the PEDOT-NG nanoarchitecture thin films was evaluated by monitoring the amperometric response in the presence of various potential interfering species at fixed potentials of 0.004, 0.160, and 0.294 V for AA, DA, and UA, respectively. Although common interferences found in biological matrices, such as glucose, cysteine, and glycine, typically compromise the selectivity of target analytes, no significant fluctuations in the response current were observed upon their addition, as depicted in Figure S9. These results indicate that the co-existing species do not interfere with the simultaneous determination of AA, DA, and UA, suggesting that the PEDOT-NG nanoarchitecture is a highly promising material for practical clinical applications. The amperometric response of PEDOT-NG nanoarchitecture thin films to target molecules such as AA, DA, and UA was highly reproducible across all four thin films, as shown in Figure S10. Consequently, the electrochemical sensing properties of the PEDOT-NG nanostructure are superior to previously reported results (see Table S2). These results confirm the suitability of the developed platform for the precise and simultaneous determination of multiple biomolecules in complex matrices.

## 4. Conclusions

We have successfully developed high-performance electrode active materials for electrochemical sensors based on PEDOT-NG and PB-NG nanoarchitectures via a one-pot electrodeposition strategy. By systematically optimizing key parameters—including applied potential, electrodeposition times, and precursor ratios—we achieved a hierarchical morphology that synergistically combines enhanced electrochemical catalytic activity with robust structural stability. The optimized PEDOT-NG nanoarchitecture demonstrated outstanding analytical performance for the simultaneous determination of AA, DA, and UA, characterized by wide linear ranges and low detection limits, while the PB-NG nanoarchitecture exhibited superior sensitivity for H_2_O_2_ detection driven by highly reversible redox kinetics. These results collectively highlight that the meticulous modulation of the interfacial micro-environment at the nanometer scale is fundamental to facilitating efficient charge transfer and expanding the electroactive surface area. Ultimately, the integration of NG as a multifunctional dopant and template offers a versatile and scalable approach for designing resilient electrode active materials. This work not only provides a comprehensive framework for next-generation biosensing but also suggests broad applicability in developing diverse electrochemical platforms for complex and demanding sensing environments.

## Data Availability

The data supporting the findings of this study are available from the corresponding author upon reasonable request, subject to privacy and legal restrictions.

## Supplementary Materials

The following supporting information can be downloaded at: https://www.mdpi.com/article/10.3390/s26082427/s1, Figure S1: Transient amperometric response at a constant potential of 0.72 V (vs. Ag/AgCl) in an aqueous solution containing K_3_[Fe(CN)_6_] (0.05 M), FeCl_3_·6H_2_O (0.05 M), and KNG (10 mg mL^−1^); Figure S2: FE-SEM images of PB-NG nanoarchitecture thin films as a function of electrodeposition cycles: (a) 1, (b) 3, (c) 5, and (d) 7 cycles, prepared at a constant potential of 0.72 V (vs. Ag/AgCl) in an aqueous solution containing 0.05 M K_3_[Fe(CN)_6_], 0.05 M FeCl_3_·6H_2_O, and 10 mg mL^−1^ KNG; Figure S3: FE-SEM images of PB-NG nanoarchitecture thin films as a function of pH of the electrodeposition solution: (a) pH 2, (b) pH 3, (c) pH 5, (d) pH 7, (e) pH 9, and (f) PB (control), fabricated at a constant potential of 0.72 V (vs. Ag/AgCl) for 5 cycles in an aqueous solution containing 0.05 M K_3_[Fe(CN)_6_], 0.05 M FeCl_3_·6H_2_O, and 10 mg mL^−1^ KNG. In the case of PB (control), 0.1 M KCl was added to the aqueous solution instead of KNG; Figure S4: UV–Vis–NIR absorption spectrum of PB-NG nanoarchitecture thin film on ITO/glass substrate; Figure S5: Plot of peak current (*I*_p_) versus the square root of scan rate in 1.0 M KCl aqueous solution; Figure S6: Reproducibility measurement of PB-NG nanoarchitecture thin films for H_2_O_2_ determination in 5 samples; Figure S7: Raman spectra characterization of the PEDOT-NG nanoarchitecture and PEDOT (control); Figure S8: Comparison of cyclic voltammograms of PEDOT-NG nanoarchitecture thin films prepared at different electrodeposition potentials. (a) −0.5~1.5 V, (b) −0.5~1.6 V, (c) −0.5~1.7 V, and (d) −0.5~1.8 V at a scan rate of 0.1 V s^−1^; Figure S9: Amperometric response of PEDOT-NG nanoarchitecture in 0.1 PBS (pH 7.4) (a) 10 μM AA, (b) 10 μM DA, and (c) 10 μM UA. (Interfering species addition is 10 μM of glucose, cysteine, and glycine); Figure S10: Reproducibility of PEDOT-NG nanoarchitecture thin film in 0.1 PBS (pH 7.4) containing 600 μM AA, 10 μM DA, and 25 μM UA; Table S1: Comparison of some characteristics of the reported electrode active materials for the determination of H_2_O_2_ [32,33,34,35]; Table S2: Comparison of some characteristics of the reported electrode active materials for the determination of AA, DA, and UA [36,37,38,39,40].
