# Supplementary material for: Optimization-Driven Engineering of Electrodeposited Nanographenide–Conductive Polymer/Prussian Blue Nanoarchitectures for Robust Electrochemical Sensing
Source: Sensors (Basel). 2026 Apr 15;26(8):2427. doi: 10.3390/s26082427 (PMC13119539; doi:10.3390/s26082427)
Supplement: Supplementary file 1 [file sensors-26-02427-s001.zip › sensors-4248881-supplementary.pdf]

Supplementary Materials for

Optimization-Driven Engineering of Electrodeposited Nanographenide–Conductive Polymer/Prussian Blue Nanoarchitectures for Robust Electrochemical Sensing

Seung Joo Jang [1], Hong Chul Lim [2,*] and Tae Hyun Kim [1,*]

[1] Department of Chemistry, Soonchunhyang University, Asan 31538, Republic of Korea
[2] Department of Pharmaceutics and Biopharmacy, Sangji University, Wonju 26339, Republic of Korea
* Correspondence: thkim@sch.ac.kr; valentie@sangji.ac.kr

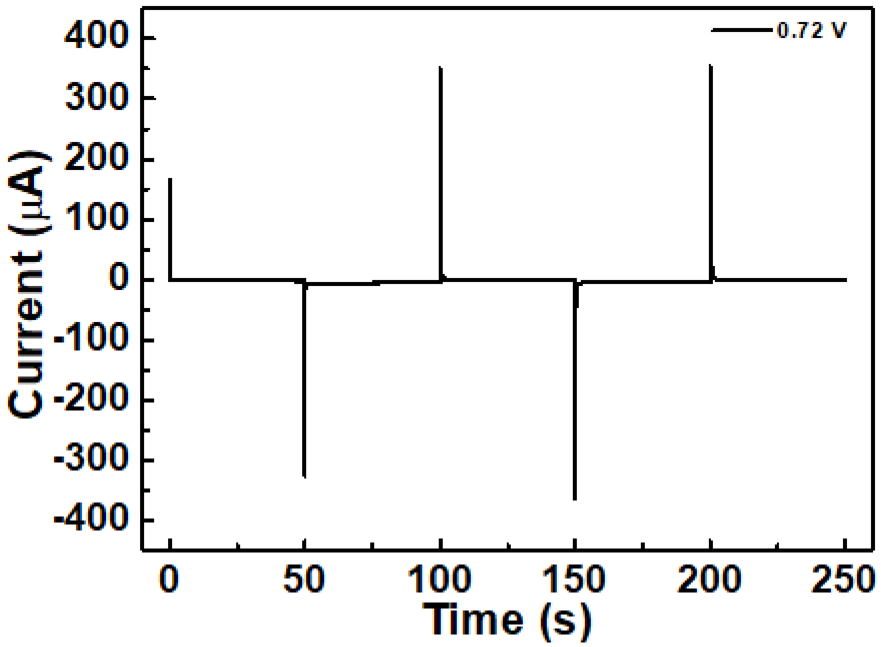

Figure S1. Transient amperometric response at a constant potential of 0.72 V (vs. Ag/AgCl) in an aqueous solution containing $K_3[Fe(CN)_6]$ (0.05 M), $FeCl_3{\cdot}6H_2O$ (0.05 M), and KNG (10 mg mL^{-1}).

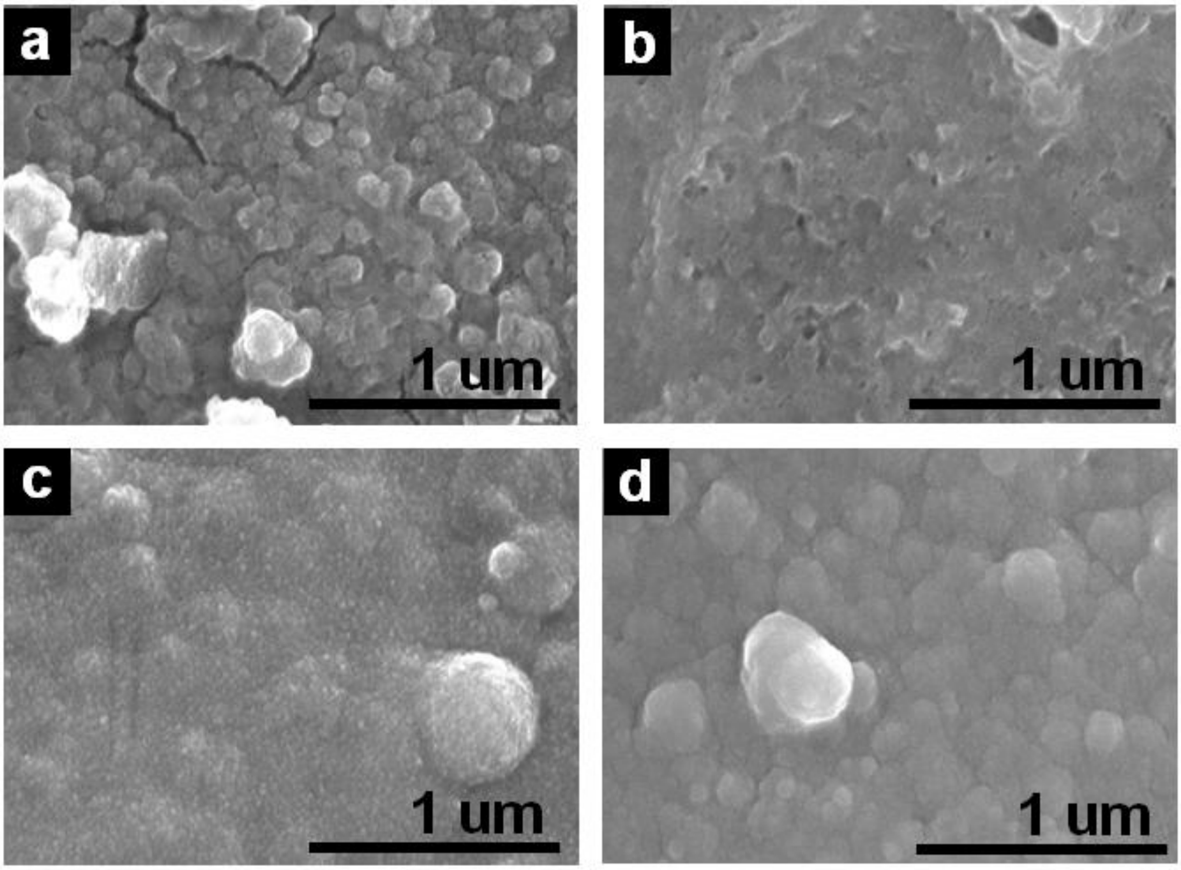

Figure S2. FE-SEM images of PB-NG nanoarchitecture thin films as a function of electrodeposition cycles: (a) 1, (b) 3, (c) 5, and (d) 7 cycles, prepared at a constant potential of 0.72 V (vs Ag/AgCl) in an aqueous solution containing 0.05 M $K_3[Fe(CN)_6]$, 0.05 M $FeCl_3 \cdot 6H_2O$, and 10 mg mL^{-1} KNG.

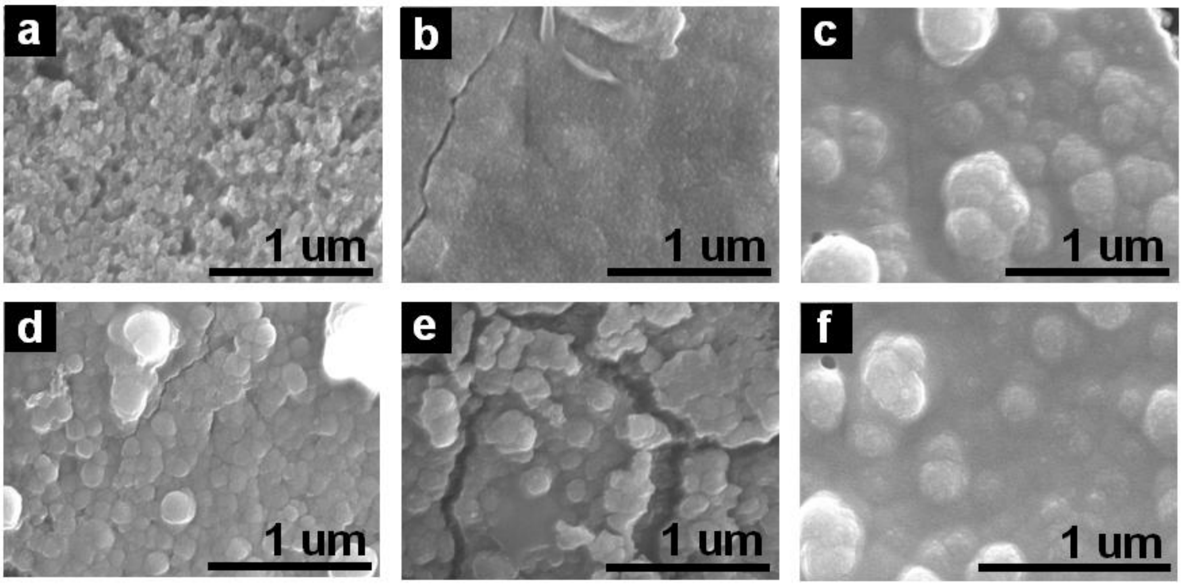

Figure S3. FE-SEM images of PB-NG nanoarchitecture thin films as a function of pH of the electrodeposition solution: (a) pH 2, (b) pH 3, (c) pH 5, (d) pH 7, (e) pH 9, and (f) PB (control), fabricated at a constant potential of 0.72 V (vs Ag/AgCl) for 5 cycles in an aqueous solution containing 0.05 M $K_3[Fe(CN)_6]$, 0.05 M $FeCl_3{\cdot}6H_2O$, and 10 mg mL^{-1} KNG. In the case of PB (control), 0.1 M KCl was added to the aqueous solution instead of KNG.

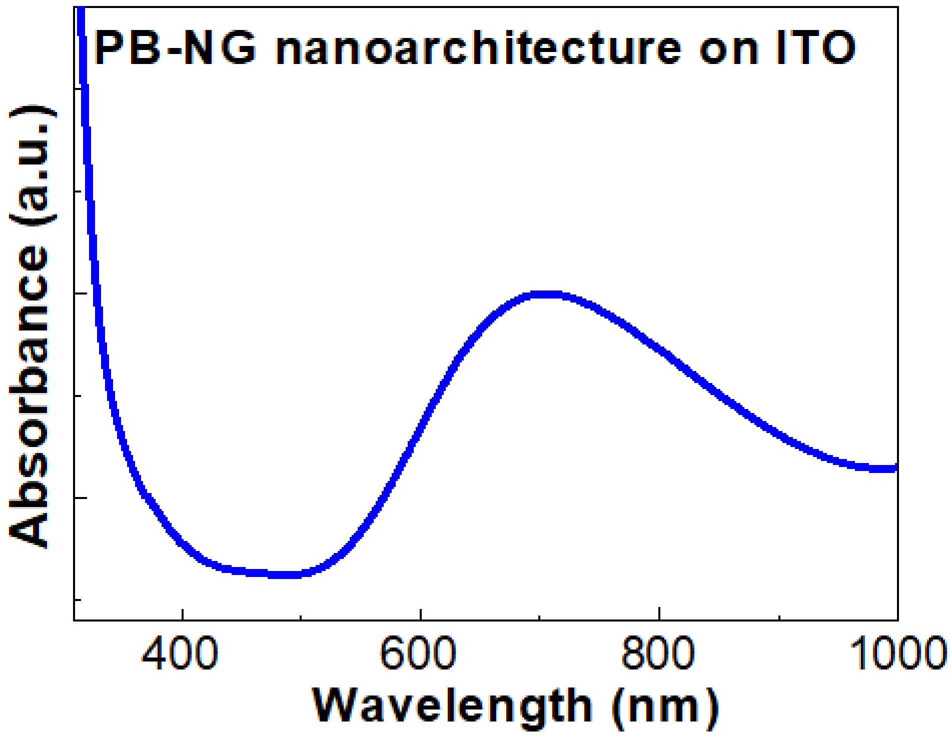

Figure S4. UV–Vis–NIR absorption spectrum of PB-NG nanoarchitecture thin film on ITO/glass substrate.

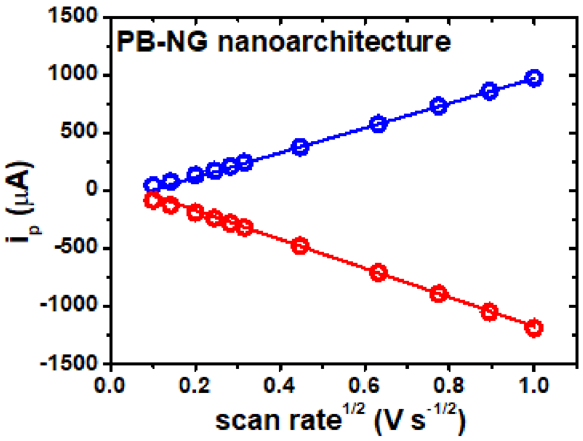

Figure S5. Plot of peak current (I_P) versus the square root of scan rate in 1.0 M KCl aqueous solution.

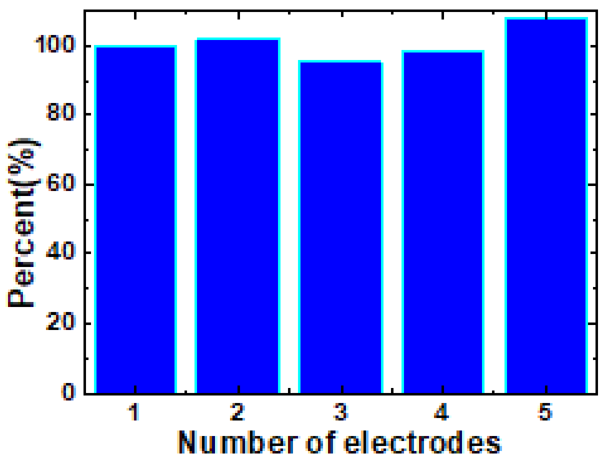

Figure S6. Reproducibility measurement of PB-NG nanoarchitecture thin films for H_2O_2 determination in 5 samples.

Table S1. Comparison of some characteristics of the reported electrode active materials for the determination of H_2O_2.

H_2O_2 Sensor	Linear range (mM)	LOD (μM)	Ref
PBNPs	0.26–1	700	[32]
PB/Polypyrrole	0.005–2.775	1.6	[33]
PEDOT-PB NPs	0.005–1	1.4	[34]
PBNPs/Nafion	0.021–0.14	1	[35]
PB-GQD	0.01–2	4.39	This work

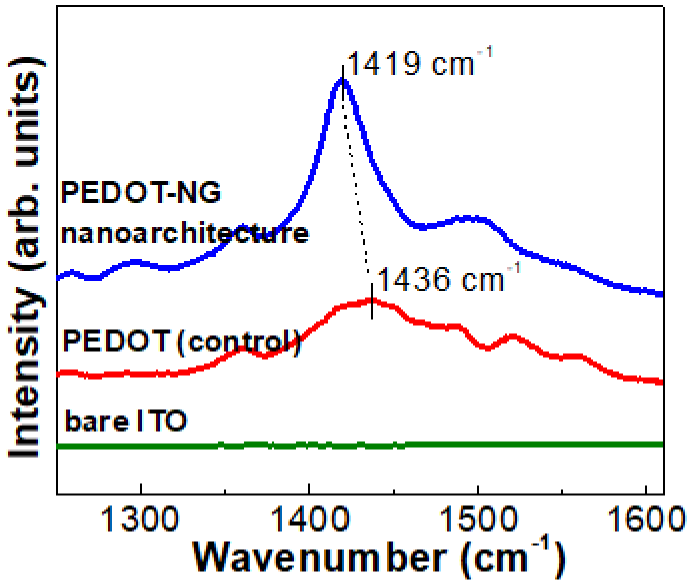

Figure S7. Raman spectra characterization of the PEDOT-NG nanoarchitecture and PEDOT (control).

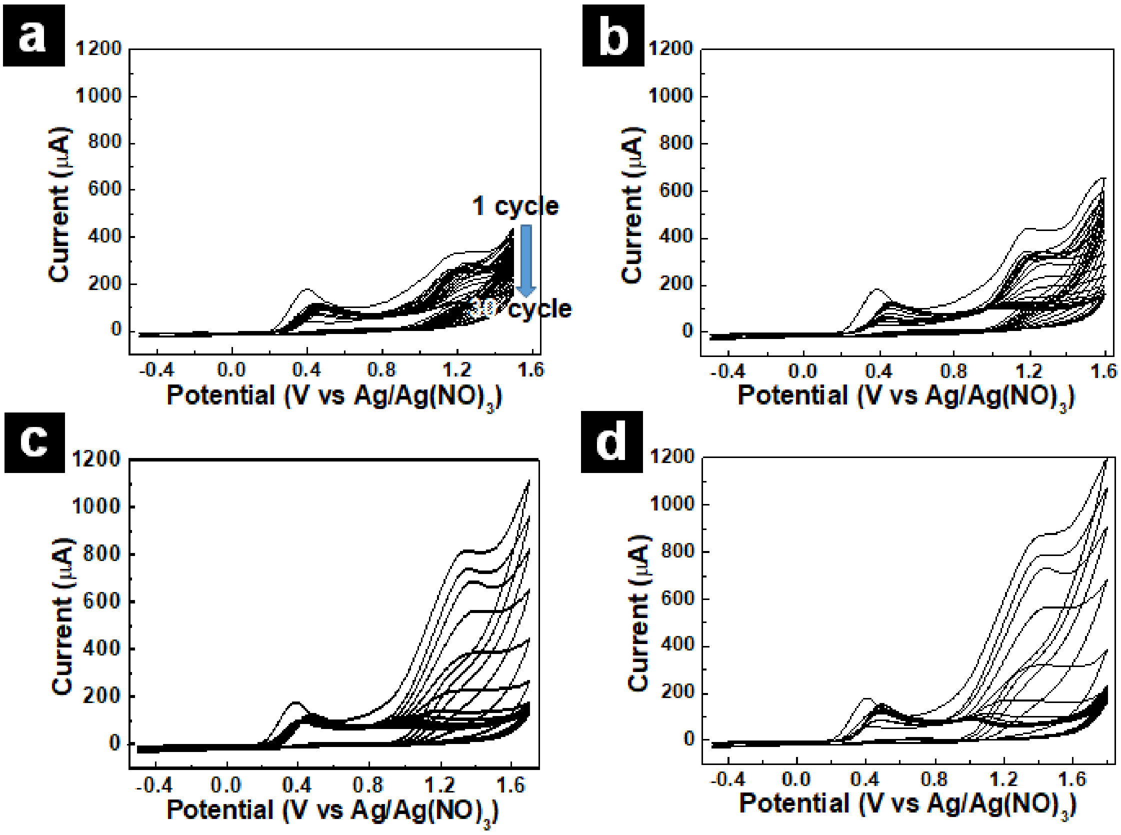

Figure S8. Comparison of cyclic voltammograms of PEDOT-NG nanoarchitecture thin films prepared at different electrodeposition potentials. (a) −0.5~1.5 V, (b) −0.5~1.6 V, (c) −0.5~1.7 V, and (d) −0.5~1.8 V at a scan rate of 0.1 V s^{-1}.

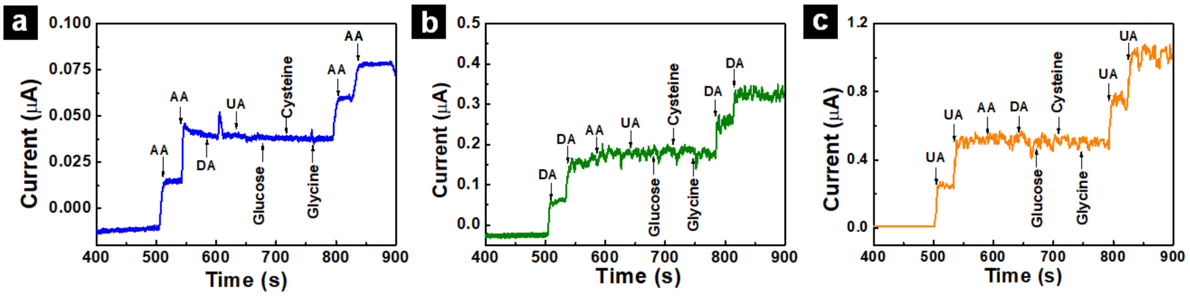

Figure S9. Amperometric response of PEDOT-NG nanoarchitecture in 0.1 PBS (pH 7.4) (a) 10 µM AA, (b) 10 µM DA, and (c) 10 µM UA. (Interfering species addition is 10 µM of glucose, cysteine, and glycine).

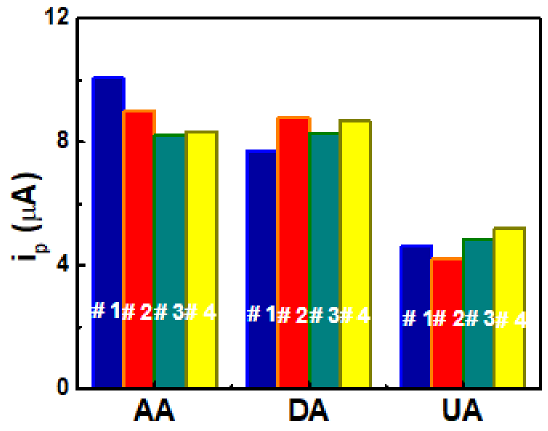

Figure S10. Reproducibility of PEDOT-NG nanoarchitecture thin film in 0.1 PBS (pH 7.4) containing 600 μM AA, 10 μM DA, and 25 μM UA.

Table S2. Comparison of some characteristics of the reported electrode active materials for the determination of AA, DA, and UA.

Material	Method	Linear range (μM)			Detection limit (μM)			Ref
		AA	DA	UA	AA	DA	UA	
MoS_2/PEDOT-GCE	DPV	20-140	1-80	2-25	5.83	0.52	0.95	[36]
MoS_2/PANI/rGO-GCE	DPV	50-8000	5-500	1-500	22.2	0.7	0.36	[37]
PEDOT/rGO/MnO_2	DPV	1-800	0.03-45	0.3-80	1.00	0.02	0.05	[38]
PEDOT-Ni/Si MCP	DPV	20-1400	12-48	36-216	10	1.5	2.7	[39]
MWCNT/PEDOT-FAD	LSV	400-8000	6-75	2-40	400	6	2	[40]
PEDOT/GQD-GCE	DPV	30-1000	0.5-40<br>40-200	1-100	4.1	0.12	0.18	This work

References

(32) Razmi, H.; Mohammad-Rezaei, R.; Heidari, H. Self-assembled Prussian blue nanoparticles based electrochemical sensor for high sensitive determination of H_2O_2 in acidic media, *Electroanalysis* **2009**, *21*, 2355–2362.

(33) Yang, Z.; Zheng, X.; Zheng, J. Facile synthesis of Prussian blue/hollow polypyrrole nanocomposites for enhanced hydrogen peroxide sensing. *Ind. Eng. Chem. Res.* **2016**, *55*, 12161–12166.

(34) Lete, C.; Marin, M.; Anghel, E. M.; Preda, L.; Matei, C.; Lupu, S. Sinusoidal voltage electrodeposition of PEDOT-Prussian blue nanoparticles composite and its application to amperometric sensing of H2O2 in human blood, *Mater. Sci. Eng. C* **2019**, *102*, 661-669.

(35) Haghighi, B.; Hamidi, H.; Gorton, L. Electrochemical behavior and application of Prussian blue nanoparticle modified graphite electrode, *Sens. Actuators B Chem.* **2010**, *147*, 270–276.

(36) Li, Y.; Lin, H.; Peng, H.; Qi, R.; Luo, C. A glassy carbon electrode modified with MoS_2 nanosheets and poly (3,4-ethylenedioxythiophene) for simultaneous electrochemical detection of ascorbic acid, dopamine and uric acid. *Microchim Acta* **2016**, *183* (9), 1–7.

(37) Li, S.; Ma, Y.; Liu, Y.; Xin G.; Wang, M.; Zhang, Z.; Liu, Z. Electrochemical sensor based on a three dimensional nanostructured MoS_2 nanosphere- PANI/reduced graphene oxide composite for simultaneous detection of ascorbic acid, dopamine, and uric acid. *RCS Adv.* **2019**, *9*, 2997–3003.

(38) Tukimin, N.; Abdullah, J.; Sulaiman, Y. Electrodeposition of poly(3,4-ethylenedioxythiophene)/reduced graphene oxide/manganese dioxide for simultaneous detection of uric acid, dopamine and ascorbic acid. *J. Electroanal. Chem.* **2018**, *820*, 74–81.

(39) Yu, S.; Luo, C.; Wang, L.; Peng, H.; Zhu, Z. Poly (3 4-ethylenedioxythiophene)modified Ni/silicon microchannel plate electrode for the simultaneous determination of ascorbic acid, dopamine and uric acid, *Analyst* **2013**, *138*, 1149–1155.

(40) Lin, K.C.; Huang, J.Y.; Chen, S.M. Simultaneous determination of ascorbic acid, dopamine, uric acid and hydrogen peroxide based on co-immobilization of PEDOT and FAD using multi-walled carbon nanotubes, *Anal. Methods* **2014**, *6*, 8321–8327.
